# Results of MyPlan 2.0 on Physical Activity in Older Belgian Adults: Randomized Controlled Trial

**DOI:** 10.2196/13219

**Published:** 2019-10-07

**Authors:** Delfien Van Dyck, Karel Herman, Louise Poppe, Geert Crombez, Ilse De Bourdeaudhuij, Freja Gheysen

**Affiliations:** 1 Department of Movement and Sports Sciences Faculty of Medicine and Health Sciences Ghent University Ghent Belgium; 2 Research Foundation – Flanders Brussels Belgium; 3 Department of Experimental Clinical and Health Psychology Faculty of Psychology and Educational Sciences Ghent University Ghent Belgium; 4 Department of Educational Policy Ghent University Ghent Belgium; 5 Department of Research and Valorisation Vives University of Applied Sciences Kortrijk Belgium

**Keywords:** self-regulation, exercise, elderly, eHealth

## Abstract

**Background:**

The beneficial effects of physical activity (PA) for older adults are well known. However, few older adults reach the health guideline of 150 min per week of moderate-to-vigorous PA (MVPA). Electronic health (eHealth) interventions are effective in increasing PA levels in older adults in the short term but, rarely, intermediate-term effects after a period without the support of a website or an app have been examined. Furthermore, current theory-based interventions focus mainly on preintentional determinants, although postintentional determinants should also be included to increase the likelihood of successful behavior change.

**Objective:**

This study aimed to investigate the effect of the theory-based eHealth intervention, MyPlan 2.0, focusing on pre- and postintentional determinants on both accelerometer-based and self-reported PA levels in older Belgian adults in the short and intermediate term.

**Methods:**

This study was a randomized controlled trial with three data collection points: baseline (N=72), post (five weeks after baseline; N=65), and follow-up (three months after baseline; N=65). The study took place in Ghent, and older adults (aged ≥65 years) were recruited through a combination of random and convenience sampling. At all the time points, participants were visited by the research team. Self-reported domain-specific PA was assessed using the International Physical Activity Questionnaire, and accelerometers were used to objectively assess PA. Participants in the intervention group got access to the eHealth intervention, MyPlan 2.0, and used it independently for five consecutive weeks after baseline. MyPlan 2.0 was based on the self-regulatory theory and focused on both pre- and postintentional processes to increase PA. Multilevel mixed-models repeated measures analyses were performed in R (R Foundation for Statistical Computing).

**Results:**

Significant (borderline) positive intervention effects were found for accelerometer-based MVPA (baseline−follow-up: intervention group +5 min per day and control group −5 min per day; *P*=.07) and for accelerometer-based total PA (baseline−post: intervention group +20 min per day and control group −24 min per day; *P*=.05). MyPlan 2.0 was also effective in increasing self-reported PA, mainly in the intermediate term. A positive intermediate-term intervention effect was found for leisure-time vigorous PA (*P*=.02), moderate household-related PA (*P*=.01), and moderate PA in the garden (*P*=.04). Negative intermediate-term intervention effects were found for leisure-time moderate PA (*P*=.01) and cycling for transport (*P*=.07).

**Conclusions:**

The findings suggest that theory-based eHealth interventions focusing on pre- and postintentional determinants have the potential for behavior change in older adults. If future studies including larger samples and long-term follow-up can confirm and clarify these findings, researchers and practitioners should be encouraged to use a self-regulation perspective for eHealth intervention development.

**Trial Registration:**

Clinicaltrials.gov NCT03194334; https://clinicaltrials.gov/ct2/show/NCT03783611.

## Introduction

### Background

The beneficial effects of physical activity (PA) for older adults (aged ≥65 years) are well known. PA reduces the risk of developing common chronic diseases, such as type 2 diabetes, cardiovascular diseases, and hypertension. In addition, PA has a positive effect on overall physical and mental functioning and on morbidity and mortality rates [1-5]. However, many older adults are not sufficiently active [3]. The World Health Organization (WHO) states that “older adults should do at least 150 min of moderate-intensity aerobic PA or 75 min of vigorous-intensity aerobic PA throughout the week, or a combination of both.” [6]. However, depending on the country, 60% to 70% of older adults in Western countries do not reach the PA health guideline [7]. Similarly, only 31% of older Belgian adults aged between 65 and 74 years are sufficiently physically active [8]. In those aged 75 years and older, this is only 12% [8]. Given these low levels of PA, it is necessary to develop effective interventions for this particular age group [9].

Overall, health behavior interventions are often not theory based. Nonetheless, it has been shown that the use of a theoretical framework for intervention development enhances the effectiveness of an intervention [10,11]. For example, the theoretical framework of self-regulation is useful for intervention development [12]. Self-regulation is defined as “a goal-guidance process aimed at the attainment and maintenance of personal goals” [12]. The process of behavior change can be divided in a pre- and a postintentional phase. In the preintentional phase, an individual acknowledges a problem (eg, the lack of PA) and develops intentions to solve this problem. In the postintentional phase, an individual sets goals and makes action plans to achieve them. In the past, interventions to increase PA levels in older adults that made use of a theoretical framework primarily targeted preintentional determinants (eg, attitude, self-efficacy, and expected outcomes) of PA [13]. However, changing these determinants does not necessarily imply that people will change their actual behavior. This is the so-called *intention behavior gap* [14]. To achieve actual behavior change, postintentional determinants (eg, making action plans and engaging in goal pursuit and goal adaptation) must also be integrated in an intervention. By focusing on the whole process of behavior change, the likelihood of successful behavior change increases.

In the last decade, researchers started using mobile apps and websites to promote PA and well-being in different age groups [15,16]. One of the major advantages of this evolution is the increasing accessibility of health care. In addition, face-to-face contact is no longer needed, and tailored interventions can be executed at home [17]. Furthermore, delivering electronic health (eHealth) interventions is less expensive than providing traditional interventions [18]. Research also indicated that eHealth interventions are suitable for older adults [19]. In 2015, The Federal Public Service of economy of Belgium reported that approximately 73% of adults aged 65 to 74 years used the internet on a daily basis. As this percentage is still increasing, eHealth interventions become more and more appropriate to promote PA in older adults.

Previous studies already showed that a tailored eHealth intervention, based on the self-regulation theory, could increase PA in (older) adults [15,16,20]. Degroote et al [20] and Plaete et al [16] showed that *MyPlan 1.0*, a website based on the self-regulation theory and the Health Action Process Approach (a specific model of self-regulation [21]), was effective in increasing PA levels in adults after a month of intervention. Similar effects were found in older adults [15].

Despite the promising results of the previous *MyPlan 1.0* intervention studies, several research questions remain unanswered. In the 3 studies mentioned above [15,16,20], assessments took place a week and a month after the start of the intervention, that is, after a period of continuous website support. On the basis of this protocol, it is impossible to determine whether these effects last for a longer period, especially when support from the website is no longer being provided. Overall, such evidence is still lacking [22]. As it is important to maintain a physically active lifestyle [23], this study will focus on the effects of the eHealth website, *MyPlan 2.0*, on PA levels in older adults after a period of 2 months without website support.

### Objective

Furthermore, previous studies mainly used self-reported PA data that are known to be subject to recall bias and over-reporting [24]. To overcome this problem, this study combines self-reported and objective methods to assess PA. Consequently, the aim of this study was to examine the short- and intermediate-term effects of the *MyPlan 2.0* eHealth intervention on objectively measured and self-reported PA levels in older adults. It was expected that self-reported and objectively measured PA levels would increase in the intervention group immediately after using the website for 5 weeks (short-term effects), compared with the control group. As the intervention was based on self-regulation, that is, guiding individuals gradually toward their goals [12,16], it was also expected that the short-term effects would be maintained in the intermediate term, after a period without website support.

## Methods

### Study Design

This study was a parallel randomized controlled trial (1:1 allocation) using random sampling in combination with convenience sampling.

#### Research Site

The study took place in Ghent and its suburbs. Ghent is the second largest city in Flanders, which is the Dutch-speaking part of Belgium. It has approximately 260,000 inhabitants.

#### Procedure

First, the Public Service of Ghent provided names and addresses of 1000 randomly selected adults aged between 65 and 80 years. Second, the research team randomly sent 500 invitation letters to participate in the *MyPlan 2.0* intervention and 500 invitation letters to be part of a control group receiving no intervention. As the response rate was very low, the research team additionally recruited participants by handing out flyers in local service centers (ie, convenience sampling).

The inclusion criteria for this study were the following: being aged 65 to 80 years, retired, able to walk 100 m without any help (ie, devices or help from persons), Dutch-speaking, and have an email address. The email address was needed for logging in to the website and for sending the weekly reminders to visit the website. Eligible participants were asked to confirm their participation by email or phone. Afterward, participants received an email with extra information about the study.

**Figure 1:**
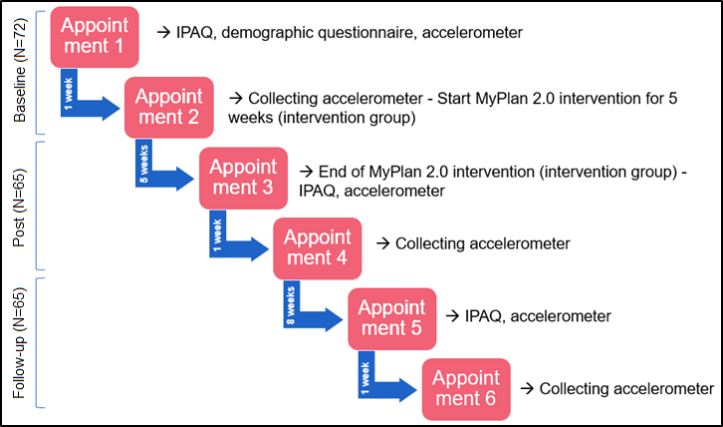
Study design of MyPlan 2.0. IPAQ: International Physical Activity Questionnaire.

**Figure 2:**
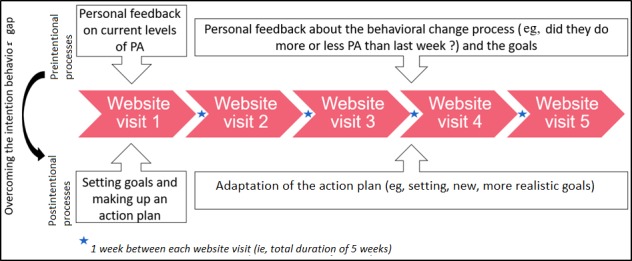
Overview of the electronic health intervention MyPlan 2.0. PA: physical activity.

The study comprised 6 appointments in person (Figure 1). Data were collected from November 2016 to June 2017. During the first appointment (baseline data collection), all participants signed the informed consent, filled out the long International Physical Activity Questionnaire (IPAQ, interview version) and a demographic questionnaire, and received an accelerometer (Figure 2). At least one week later (appointment 2), the accelerometer was recollected and participants of the intervention group were invited to use the *MyPlan 2.0* intervention for 5 consecutive weeks (ie, 5 website visits). The control group did not get access to the website. After 5 weeks, when the intervention group completed the *MyPlan 2.0* intervention, the post data collection took place: participants were interviewed (long IPAQ) and asked to wear the accelerometer for the second time (appointment 3). A week later, the accelerometer was recollected (appointment 4). A total of 3 months after baseline, follow-up measurements were conducted. The participants wore the accelerometer and filled out the IPAQ (interview) for the last time (appointment 5). A week later, during the final appointment, the accelerometer was recollected. The study protocol was approved by the Ethics Committee of the Ghent University Hospital (project number 2015/1502).

### Intervention

In this study, *MyPlan 2.0*, an improved version of *MyPlan 1.0* [20,21], was used. *MyPlan 1.0* was mainly theory based, whereas *MyPlan 2.0* is theory- and user-based. Several qualitative studies were performed to optimally adapt the intervention to the users’ needs. For example, users of *MyPlan 1.0* indicated that they felt demotivated by the extensive questionnaires they had to complete to receive tailored feedback and stated that they did not understand why creating coping plans would help alter their behavior [25]. In *MyPlan 2.0*, these questionnaires were significantly shortened and rationales for the implemented behavior change techniques were added. Moreover, Vandelanotte et al [26] showed that interventions with minimum 5 contact moments (eg, appointments, Web modules, and emails) were more successful. Therefore, *MyPlan 2.0* comprised 5 website visits in contrast to the 3 obligatory website visits of *MyPlan 1.0*. The first website visit (for details see below) contained pre- and postintentional processes. The following 4 website visits mainly contained postintentional processes. Participants could independently use the website, without researcher involvement.

During the first website visit, participants had to complete a short PA questionnaire and based on the answers, they received computer-tailored or personalized feedback. By doing so, the preintentional processes were targeted (Figure 2). This personalized feedback was based on a comparison of the users’ PA levels with the health guidelines of 150 min per week of moderate-to-vigorous PA (MVPA) [6]. To increase knowledge, users had the option to complete a quiz about PA and its beneficial effects. As shown in Figure 2, postintentional processes were targeted by asking the participants to make an action plan. By doing so, the gap between intentions and behavior was bridged. Participants were asked *what* they wanted to do (eg, being more active by cycling during leisure), *when* (eg, every Sunday morning), *where* (eg, in the streets nearby), and *for how long* (eg, 60 min) they were planning to do the activity. After providing answers to these questions, participants could identify difficult situations and possible barriers (ie, coping planning) while pursuing their goals, using a predefined list of situations and barriers. Depending on which barriers they selected, specific solutions were given, and participants could choose which ones they considered most appropriate and applicable. At the end of this first website visit, users could indicate how they wanted to self-monitor their behavior (eg, using an agenda), and they could read more information about how to receive support toward PA from their social environment. Finally, the personal action plan could be printed weekly (optional). Multimedia Appendix 1 provides screenshots from the website and links these to the self-regulation techniques that were used.

A week after finishing the first website visit, participants received an email to revisit the website. During this second visit, they received feedback about their behavioral change process and goals (eg, *did you reach your goal or not?*). Afterward, participants had the possibility to adapt their action plan (eg, setting new, more realistic goals) and reconsider coping plans based on the barriers they experienced while pursuing their goals. Furthermore, participants could optionally read tips on how to increase PA.

Website visits 3, 4, and 5 were respectively activated 1 week after the previous visit. Again, participants were reminded by email. These 3 last visits were identical to the second visit (reviewing the action and coping plans). If participants did not revisit the website after 1 week, the research team phoned them reminding them to revisit the website. Figure 2 provides an overview of the intervention.

### Participants

**Figure 3:**
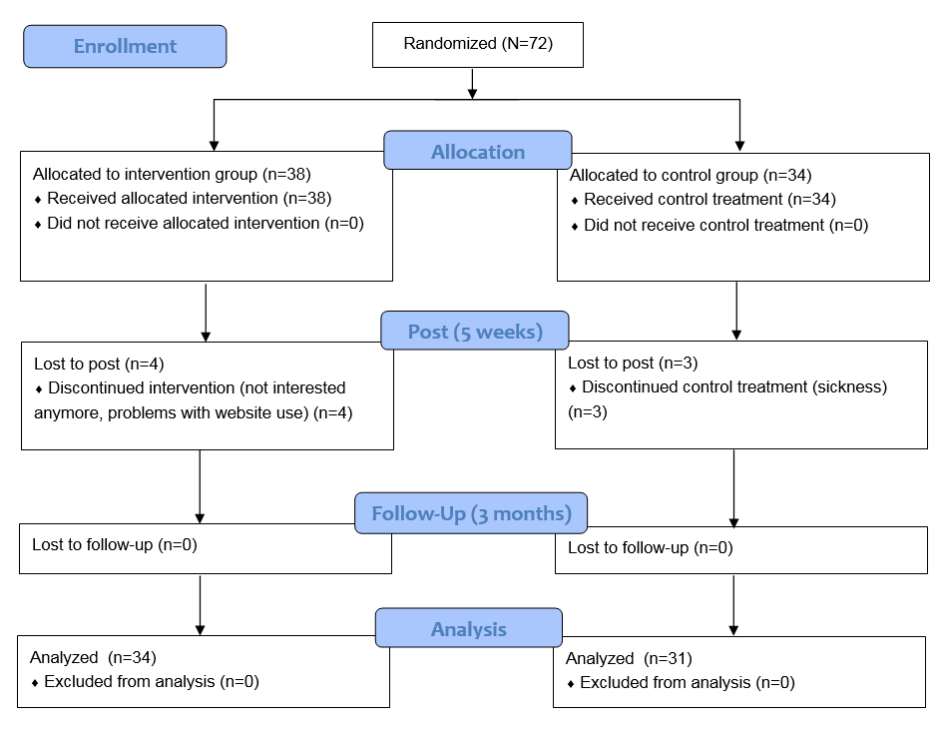
Participant flow diagram.

At baseline, the total sample comprised 72 older adults, 38 in the intervention group and 34 in the control group. Between pre and post measurements, 7 people dropped out. Of them, 3 were part of the control group and 4 of the intervention group. Reasons for dropping out were as follows: no longer interested in the intervention (n=2), sickness (n=3), and problems with using the website (n=2). There was no dropout between post and follow-up measurements (Figure 3).

### Instruments and Materials

The following sociodemographic variables were assessed at baseline: age, gender, height and weight, marital status (married, widowed, divorced, cohabiting, and living alone), and highest degree of education (primary school, secondary school, college, and university).

Self-reported PA was assessed at baseline, post, and follow-up, using the long Dutch IPAQ interview version (usual week version). This questionnaire assesses the frequency and duration of walking, cycling, moderate-intensity PA, and vigorous-intensity PA in 4 domains: (voluntary) work, transport, leisure, and household (home and garden). The IPAQ has good reliability (intraclass range from 0.46-0.96), and the criterion validity is fair-to-moderate with Spearman rho ranging from 0.30 to 0.37 [27,28]. As the IPAQ has a tendency of over-reporting, all data were truncated according to the official IPAQ guidelines [29].

Objective PA was assessed at baseline, post, and follow-up, using an ActiGraph GT3X+ accelerometer. Participants wore the accelerometer for 7 days on the right hip. They were asked to wear it during waking hours but not when swimming, showering, or practicing a contact sport. The accelerometers were initialized and processed using Actilife 6.13.3. Valid wear time was set as at least four days with at least ten hours of wear time. Non–wear time was defined as ≥60 min of consecutive zeros. The epoch was set at 60 seconds, and the cut point used to determine MVPA was set at 1952 counts per minute (cpm) [30]. The cut point for light-intensity activity was set at 100 to 1951 cpm. The level of total PA was calculated by adding up light-intensity PA and MVPA.

### Statistical Analyses

Baseline sociodemographic characteristics of the intervention and control group were compared using independent sample *t* tests (continuous variables) and chi-square tests (categorical variables) in SPSS 25.0. To evaluate the intervention effects on accelerometer-assessed and self-reported PA, multilevel mixed-models repeated measures analyses were performed in R (package lme4) [31]. Multilevel modeling (2-level: measurement–participant) was applied to take into account the clustering of the 3 measurements (pre–post–follow-up) in participants. On the basis of the recommendations of Chakraborty and Gu [32], no ad hoc data imputation was applied. As almost all PA variables, except for accelerometer-based total PA, were positively skewed, square-root transformations were applied to improve normality. To increase the comprehensibility of the tables, raw descriptive data have been reported, although analyses were conducted using the square-root transformed data. For each PA variable (2 accelerometer-based and 11 self-reported PA variables), a separate regression model was fitted. The reported beta value for the interaction effect between *time* and *condition* can be interpreted as the difference in change in outcome between pre- and posttest, pre- and follow-up test, and post- and follow-up test according to the condition to which participants belong (intervention vs control condition). Statistical significance was set at *P*<.05 but because of the small sample size, borderline significant results (*P*<.10) were also reported.

## Results

### Participants

Baseline descriptive statistics are shown in Table 1. At baseline, 72 older adults (38 in intervention group and 34 in control group) participated in this study, 51% (37/72) were male. The participants’ mean age was 70.9 (SD 4.1) years, and mean body mass index was 26.4 (SD 4.2) g/m^2^. In total, 64% (46/72) of all participants were married, and 47% (34/72) had a college or university degree. There were no significant baseline differences in sociodemographic characteristics between the intervention and the control group. Consequently, no covariates were included in further analyses.

**Table 1:** Sociodemographic characteristics at baseline.

Sociodemographic characteristics		Total sample (N=72)	Control group (n=34)	Intervention group (n=38)	*X*² value (*df*)
Age (years), mean (SD)		70.9 (4.1)	70.9 (4.1)	70.8 (4.1)	0.1 (*70*)^a^
**Gender, n (%)**					
	Male	37 (51)	19 (56)	18 (47)	0.5 (*1*)
	Female	35 (49)	15 (44)	20 (53)	—^b^
**Educational level, n (%)**					
	No college/university	38 (53)	16 (47)	22 (58)	0.9 (*1*)
	College/university	34 (47)	18 (53)	16 (42)	—
**Marital status, n (%)**					
	Married	46 (64)	24 (71)	22 (58)	3.4 (*1*)
	Not married	26 (36)	10 (29)	16 (42)	—
Body mass index, mean (SD)		26.4 (4.2)	26.8 (4.2)	26.0 (4.2)	0.8 (*70*)^a^

^a^*t* values with df.

^b^Not applicable.

### Intervention Effects on Accelerometer-Based Physical Activity Levels

Results of the multilevel mixed-models repeated measures analyses for accelerometer-based PA are shown in Table 2. A borderline significant intervention effect between baseline and post was found for accelerometer-assessed total PA (*P*=.07). Participants in the intervention group increased their total PA, whereas those in the control group had a decrease in total PA between baseline and post. Similarly, the intervention effect (baseline−follow-up) was borderline significant for accelerometer-based MVPA (*P*=.07); accelerometer-based MVPA increased in the intervention and decreased in the control group.

**Table 2:** Intervention effects (time-by-group interactions) for objectively assessed physical activity levels (participants with valid accelerometer data in intervention group: baseline=35, post=31, follow-up=32 and control group: baseline=31, post=30, follow-up=27).

Dependent variables (minutes/day)		Baseline (N=66), mean (SD)	Post (N=61), mean (SD)	Follow-up (N=59), mean (SD)	Group×time			
					Reference = Control×pre, beta (SE)	*P* value	Reference = Control×post, beta (SE)	*P* value
**Total physical activity (minutes/day)**								
	Control	283.9 (85.2)	259.8 (71.4)	259.8 (71.4)	Post: 39.4 (19.9)	.05	Follow-up: −24.6 (15.9)	.13
	Intervention	273.3 (70.4)	293.7 (85.4)	288.9 (77.5)	Follow-up: 6.5 (6.9)	.35	—^a^	—
**Moderate-to-vigorous physical activity (minutes/day)^b^**								
	Control	29.9 (38.0)	22.1 (14.1)	24.0 (18.3)	Post: 0.8 (0.5)	.13	Follow-up: −0.1 (0.5)	.89
	Intervention	17.6 (14.1)	25.6 (30.2)	22.9 (18.9)	Follow-up: 0.4 (0.2)	.07	—	—

^a^Not applicable.

^b^Square-root transformed.

### Intervention Effects for Self-Reported Physical Activity Levels

Results of the multilevel mixed-models repeated measures analyses for self-reported domain-specific PA levels are shown in Table 3. For leisure-time PA, (borderline) significant group×time interaction effects were found for vigorous (baseline−follow-up; *P*=.02) and moderate (baseline−post; *P*=.09 and baseline−follow-up; *P*=.01) PA. Leisure-time vigorous PA increased in the intervention and decreased in the control group. For leisure-time moderate PA, the intervention effects were inverse: participants in the control group increased their leisure-time moderate PA, whereas this increased less strongly (baseline−post) or decreased (baseline−follow-up) in the intervention group. For overall leisure-time PA, no significant intervention effects were found. For household-related PA, significant intervention effects were found for moderate PA in the garden (baseline−follow-up; *P*=.04 and post−follow-up; *P*<.001) and moderate household-related PA at home (baseline−post; *P*=.04 and baseline−follow-up; *P*=.01). All intervention effects were in the expected direction: regarding moderate PA in the garden, participants in the intervention group had a steeper increase than participants in the control group. Moderate household-related PA at home increased in the intervention and decreased in the control group. Similarly, a positive intervention effect was found for overall household-related PA (baseline−follow-up; *P*=.05). Finally, a negative intervention effect was found for cycling for transport (post−follow-up; *P*=.07; borderline significant): participants in the control group had a stronger increase in cycling for transport between post and follow-up than participants in the intervention group. For overall transport-related PA, no significant intervention effects were found.

**Table 3:** Intervention effects (time-by-group interactions) for self-reported domain-specific physical activity (number of participants in the intervention group: baseline=38, post=34, follow-up=34 and control group: baseline=34, post=31, follow-up=31).

Dependent variables (minutes/week)		Baseline (N=72), mean (SD)	Post (N=65), mean (SD)	Follow-up (N=65), mean (SD)	Group×time			
					Reference = Control×pre, beta (SE)	*P* value	Reference = Control×post, beta (SE)	*P* value
**Overall leisure-time physical activity**								
	Control	214.3 (252.3)	324.5 (295.1)	270.2 (272.6)	Post: −3.9 (3.2)	.23	Follow-up: 1.5 (3.3)	.65
	Intervention	169.9 (197.2)	220.2 (272.1)	185.9 (198.1)	Follow-up: −2.4 (3.0)	.43	—^a^	—
**Leisure-time walking^b ^**								
	Control	156.3 (214.5)	195.8 (226.0)	150.6 (193.1)	Post: −2.3 (2.3)	.32	Follow-up: 2.3 (2.3)	.31
	Intervention	92.4 (159.8)	109.6 (209.1)	103.8 (124.9)	Follow-up: −3.8 (9.6)	.97	—	—
**Leisure-time vigorous physical activity^b^**								
	Control	32.6 (146.3)	15.5 (57.9)	15.5 (86.2)	Post: 1.8 (1.1)	.11	Follow-up: 0.4 (1.4)	.77
	Intervention	0.00 (0.00)	30.0 (145.0)	22.1 (78.0)	Follow-up: 1.1 (0.5)	.02	—	—
**Leisure-time moderate physical activity^b^**								
	Control	25.3 (55.2)	113.2 (220.1)	104.0 (179.8)	Post: −3.6 (2.1)	.09	Follow-up: −2.1 (2.4)	.39
	Intervention	77.5 (153.1)	80.6 (137.0)	60.0 (159.8)	Follow-up: −2.9 (1.0)	.01	—	—
**Overall household-related physical activity**								
	Control	345.6 (292.2)	345.8 (328.5)	385.6 (365.4)	Post: 3.0 (3.3)	.36	Follow-up: 3.6 (3.5)	.30
	Intervention	360.8 (360.2)	414.3 (351.2)	603.2 (415.9)	Follow-up: 6.6 (3.4)	.05	—	—
**Moderate physical activity garden^b ^**								
	Control	30.8 (60.3)	74.4 (184.6)	82.3 (216.1)	Post: −2.1 (1.9)	.28	Follow-up: 6.7 (1.9)	<.001
	Intervention	32.8 (74.3)	17.6 (56.8)	150.0 (212.3)	Follow-up: 2.4 (1.1)	.04	—	—
**Vigorous physical activity garden^b^**								
	Control	17.6 (84.2)	23.2 (129.3)	61.9 (212.1)	Post: 1.5 (2.0)	.44	Follow-up: −3.2 (2.2)	.15
	Intervention	37.9 (165.3)	58.2 (168.7)	22.9 (100.2)	Follow-up: −.8 (1.0)	.40	—	—
**Moderate physical activity home^b^**								
	Control	297.1 (290.6)	248.2 (227.4)	241.5 (246.0)	Post: 4.5 (2.2)	.04	Follow-up: 3.1 (2.4)	.19
	Intervention	290.1 (309.5)	338.4 (315.6)	430.3 (328.1)	Follow-up: 3.7 (1.4)	.01	—	—
**Overall physical activity for transport**								
	Control	196.6 (228.7)	205.5 (200.8)	277.9 (327.5)	Post: −1.8 (2.8)	.52	Follow-up: −1.7 (2.7)	.52
	Intervention	190.8 (284.8)	136.8 (142.4)	153.5 (154.7)	Follow-up: −3.5 (2.9)	.24	—	—
**Walking for transport^b^**								
	Control	148.7 (180.2)	161.6 (178.6)	171.8 (217.8)	Post: −1.0 (2.2)	.66	Follow-up: −0.3 (0.9)	.88
	Intervention	133.8 (216.6)	92.9 (122.6)	99.7 (134.7)	Follow-up: −5.9 (1.1)	.59	—	—
**Cycling for transport^b^**								
	Control	47.9 (102.6)	43.9 (84.1)	106.1 (171.7)	Post: .20 (1.3)	.88	Follow-up: −2.3 (1.2)	.07
	Intervention	57.0 (137.8)	43.8 (83.2)	53.8 (91.5)	Follow-up: −1.0 (0.8)	.17	—	—

^a^Not applicable.

^b^Square-root transformed.

## Discussion

### Principal Findings

This study aimed to examine the short- and intermediate-term effects of the *MyPlan 2.0* eHealth intervention on objectively measured and self-reported PA levels in older adults. Regarding objectively measured PA, the results showed that *MyPlan 2.0* had positive but only borderline significant effects for accelerometer-based total PA in the short term and accelerometer-based MVPA in the intermediate term, when support of the website was no longer present. If our findings can be confirmed in a larger study sample, this could suggest that integrating self-regulation principles in behavior change interventions can lead to behavior change [12,33]. The intermediate-term effects found in this study are promising toward health promotion in older adults in the future. Our results are in line with a study by Irvine et al [34], showing intermediate-term effects of an eHealth intervention in adults aged older than 55 years. In that study, most of the positive intervention effects on self-reported PA were maintained after a 3-month period without support from the intervention [34]. To our knowledge, no other study previously examined whether effects of an eHealth intervention on PA in older adults remained after a period without support from the website. The positive intermediate-term effects on MVPA, in the absence of short-term effects, might be explained by the fact that self-regulation can be seen as a goal-guiding process during which individuals are gradually guided toward their goals [12,15]; it might take some time to reach these goals. In addition, it may be that participants start with increasing light-intensity PA, which is more easily achievable and can be reflected in an increase of total PA. When they feel sufficiently comfortable and ready for a *next step*, they might switch to specifically increasing MVPA after a few weeks.

From a health perspective, the effects on accelerometer-based MVPA are very promising. The WHO states that older adults should be physically active for at least 150 min per week [6]. The eHealth intervention *MyPlan 2.0* was able to increase the levels of MVPA in older adults with an average of 5 min per day between baseline and follow-up. This equals an average increase of 35 min per week, which can have a large impact on population health if the intervention would be implemented on a larger scale.

When taking a closer look at the results of objectively assessed PA, it is notable that objectively assessed MVPA decreases between baseline and follow-up in the control group, whereas the intervention group shows an increase. This may be because of seasonal effects. A study by Tucker and Gilliland [35] states that PA levels vary with seasonality. As our baseline measurements took place during an exceptionally warm autumn, post measurements during winter, and follow-up measurements during the beginning of spring (cold and rainy weather), this may be an important reason why MVPA levels decreased between baseline and follow-up in the control group. When linked to the increase in MVPA found in the intervention group, it could be that *MyPlan 2.0* might prevent the seasonal decline in MVPA that is common in older adults, as was observed in the control group. This suggests that, if this study was conducted during 1 season, a greater absolute increase in the intervention group might have been established. Evidently, this is a post hoc explanation and requires further scrutiny.

Besides the effects on objective PA data, this study also investigated the effects of *MyPlan 2.0* on self-reported domain-specific PA. Positive (borderline) significant intervention effects were found for leisure-time vigorous PA, moderate PA in the garden, and moderate household-related PA. However, inverse effects were found for leisure-time moderate PA and cycling for transport. There was no clear consistency in the timing of the positive intervention effects; most effects were found between baseline and follow-up or between post and follow-up, indicating intermediate-term effects. Again, the positive intermediate-term effects, in the absence of short-term effects, might suggest that it takes time for self-regulation techniques to be adopted and used by participants [12,14].

It is important to note that 3 intervention effects were inverse. Cycling for transport (between post and follow-up, borderline significant) and leisure-time moderate PA (both between baseline and post, borderline significant, and between baseline and follow-up, significant) increased more in the control group than the intervention group (small increase or decrease). This suggests that the intervention had a negative effect on these 2 PA domains. It is important to note that users could choose which domain they targeted in their action plans. When examining the content of the action plans in detail, it became clear that no action plans specifically focused on increasing cycling for transport and few action plans (11 out of 59) focused on leisure-time moderate PA (eg, jogging, swimming, and cycling). Of these 11 action plans, 6 focused on both moderate PA and walking. So, the increases in these behaviors in the control group, as opposed to the decreases or less steep increases in the intervention group, may be because of other reasons that remain unclear until now. A potential other reason could be that some individuals from the control group bought a new bike during the intervention period and, consequently, increased their cycling for transport and/or leisure-time moderate PA. However, this is speculative reasoning as we did not assess whether or not individuals of the control group bought a new bike.

Exploring the content of the action plans in more detail also revealed that many action plans focused on walking during leisure-time (solely or in combination with other leisure-time behaviors: 19/59) and PA at home or in the garden (15/59). This can explain the effects found on household-related PA, but, remarkably, no intervention effects were found on walking during leisure-time. This suggests that participants might not always act upon their proposed action plans. It should be noted that participants were allowed to make more than one action plan, and a previous study showed that participants who formulated multiple action plans focusing on different PA domains were not able to fulfill all these plans [36]. Of course, this is a post hoc reasoning that should be substantiated with data (eg, from personal interviews with participants) to make it possible to draw definite conclusions.

Overall, the finding that effects were mainly found in the intermediate term, when support of the website was no longer present, confirms the practical relevance of developing theory-based eHealth interventions using a self-regulatory perspective. Although our study sample was small, and the results needed to be confirmed in a trial with a longer period without website support, the findings tentatively suggest that eHealth interventions, focusing on pre- and postintentional determinants using specific behavior change techniques, have potential for behavior change in older adults. Consequently, researchers and practitioners should be encouraged to use principles of the self-regulation theory when developing eHealth interventions.

### Strengths and Limitations

The main limitation of this study was the low response rate. Of the 1000 retired adults who received a letter, only 5% participated in the study. Additional recruiting (convenience sampling) was needed until the baseline sample of 72 older adults was reached. This indicated that participants were probably very motivated to increase their PA levels, which may have biased the results. Owing to the low response rate, it is not possible to generalize these study results to the general population of older adults. Second, baseline accelerometer-based MVPA differed between the intervention and control group (average difference of 12.3 min per day). Although a multilevel analysis approach was used taking into account clustering of measurements within participants and controlling for baseline PA levels, this large baseline difference in MVPA might have influenced our results. Finally, only retired older adults were included in this study. As PA levels of retired adults might differ from those of working adults, this limits the generalizability of our findings and the comparability with other studies.

The study also had some methodological and theoretical contributions. First of all, the dropout rate was low (10%, 7/72). The study of Degroote et al [20] examining *MyPlan1.0* in adults had a dropout rate of 76% in the intervention group and 56% in the control group. In this study, this was 6.9% in the intervention group and 2.8% in the control group, respectively. This very low dropout might be explained by the improvements that were done to the *MyPlan* website and also by the fact that older adults were targeted in this study. It has been shown that older adults are less likely to drop out from studies than adults [15]. Furthermore, telephone calls were conducted to remind participants to revisit the website when this was not done timely. This might have helped to limit the dropout. However, it is important to keep in mind that follow-up telephone calls might not be feasible when the intervention would be implemented on a larger scale. This may lead to higher attrition rates. Second, objective and self-reported PA levels were measured using validated instruments. Finally, this was one of the first studies to examine the intermediate-term effects of an eHealth intervention when support from the website was no longer available.

### Recommendations for Future Research

Future research should examine whether these intervention effects last in the long term in a larger sample. Furthermore, other self-regulation interventions should aim to examine intermediate- and long-term intervention effects instead of focusing mainly on short-term effects. Ideally, the follow-up period should be extended to 6 months to 1 year. In this way, the evidence base on the intermediate- and long-term potential of eHealth interventions aiming to increase PA can be strengthened. To increase the response rate, other recruitment strategies should be used. Recruiting older adults through local service centers, community health centers, and associations for older adults may be more promising than simple random selection through a postal invitation letter. Providing small incentives can also help increase the response rate. Finally, studies using a comparable study protocol and intervention in different countries worldwide should be encouraged. Internet use in older adults differs strongly across countries [37], so it would be useful to discover whether these differences affect the effects and attrition rates of eHealth interventions.

### Conclusions

In conclusion, this study adds evidence for the effectiveness of eHealth interventions after a period without support from the website. *MyPlan 2.0* was effective in increasing self-reported leisure-time vigorous PA and moderate household-related PA (home and garden), mainly in the intermediate term when support of the website was no longer present. Although the findings for accelerometer-based MVPA were only borderline significant, this study provided a first indication of the potential of eHealth interventions to increase objectively assessed MVPA in older adults. Future studies with larger samples and long-term follow-up are needed to confirm and clarify these findings.

## Appendix

Multimedia Appendix 1 Screenshots of the MyPlan 2.0. website – implementation of self-regulation techniques.

Multimedia Appendix 2 CONSORT-eHealth checklist (V1.6.1).

## Abbreviations

cpm: counts per minute
eHealth: electronic health
FWO: Research Foundation Flanders
IPAQ: International Physical Activity Questionnaire
MVPA: moderate-to-vigorous physical activity
PA: physical activity
WHO: World Health Organization
