# Supplementary material for: Results of MyPlan 2.0 on Physical Activity in Older Belgian Adults: Randomized Controlled Trial
Source: J Med Internet Res. 2019 Oct 7;21(10):e13219. doi: 10.2196/13219 (PMC6803893; doi:10.2196/13219)
Supplement: Multimedia Appendix 1 [file jmir_v21i10e13219_app1.pdf]

Multimedia appendix 1. Screenshots of the MyPlan 2.0. website – implementation of self-regulation techniques

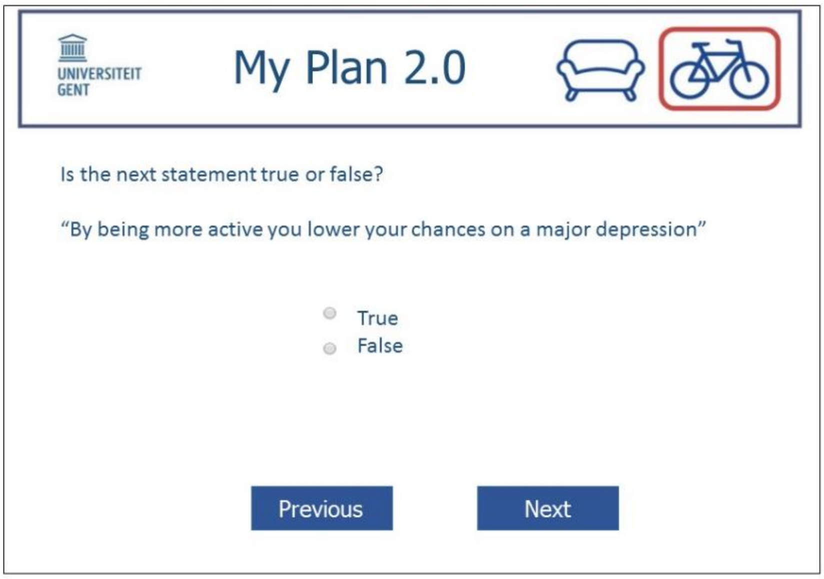

Providing information on the consequences of behaviour: the website offers the opportunity to take a quiz regarding physical activity.

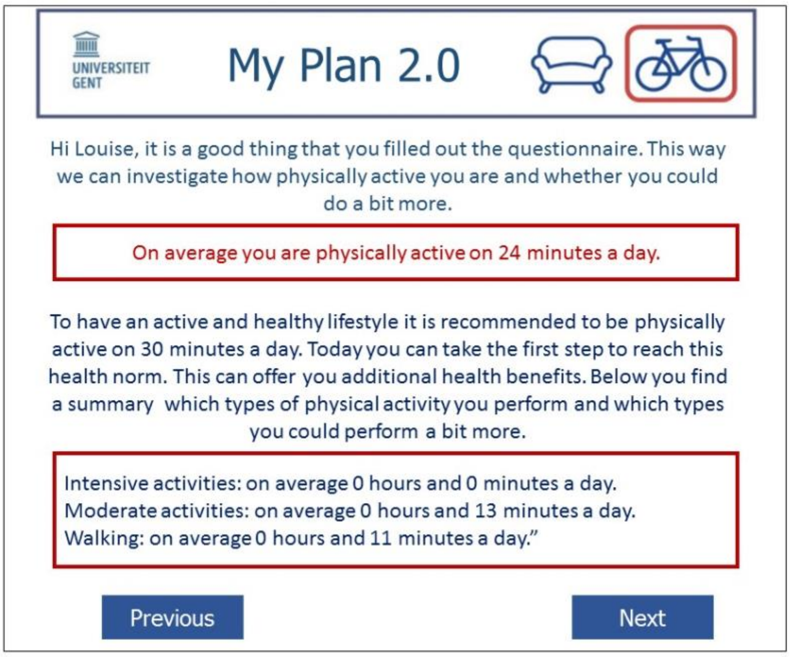

Providing feedback on performance: participants complete the short International Physical Activity Questionnaire and receive tailored feedback.

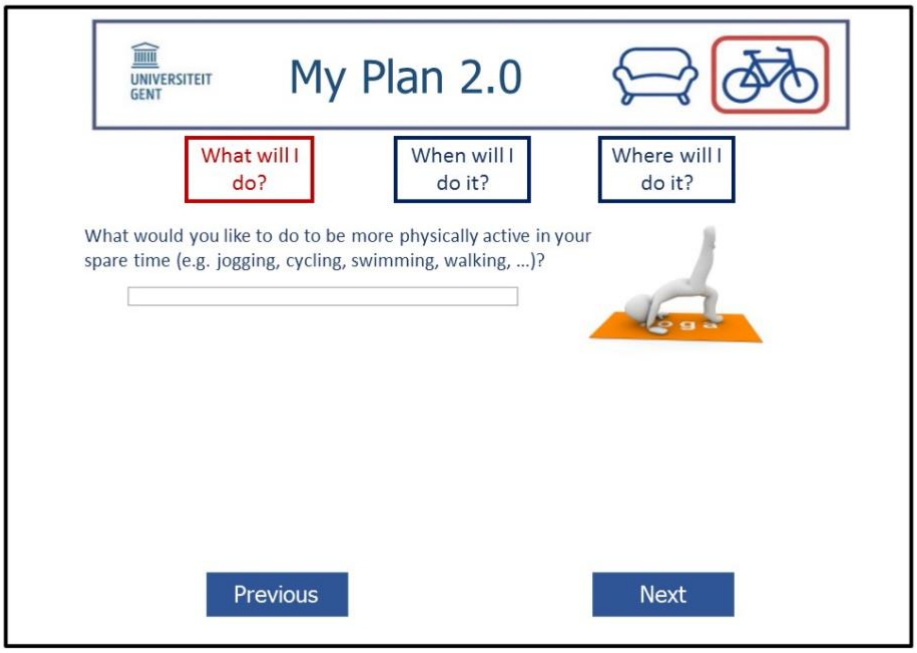

Action planning: participants specify their actions in terms of what, when and where by answering multiple questions.

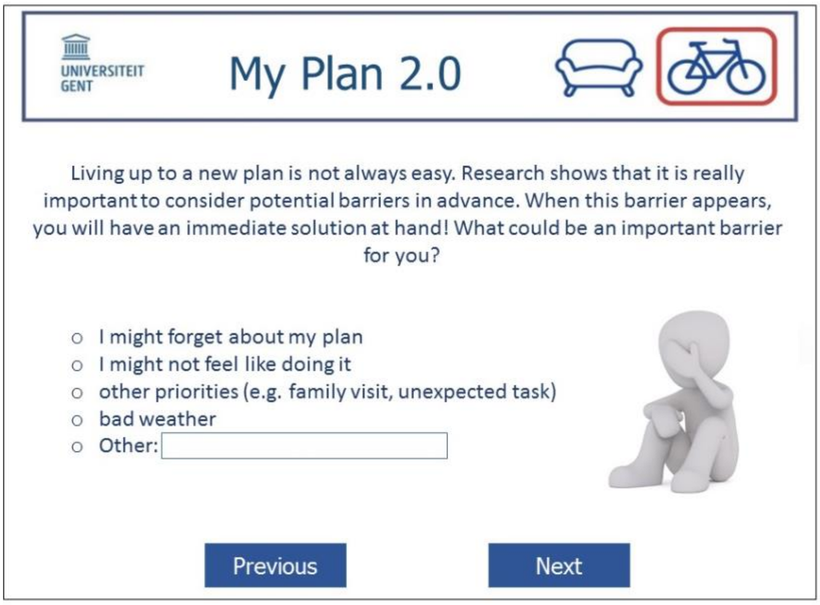

Barrier identification / problem solving: Participants define which barriers they may perceive and which solutions are possible to overcome these barriers.

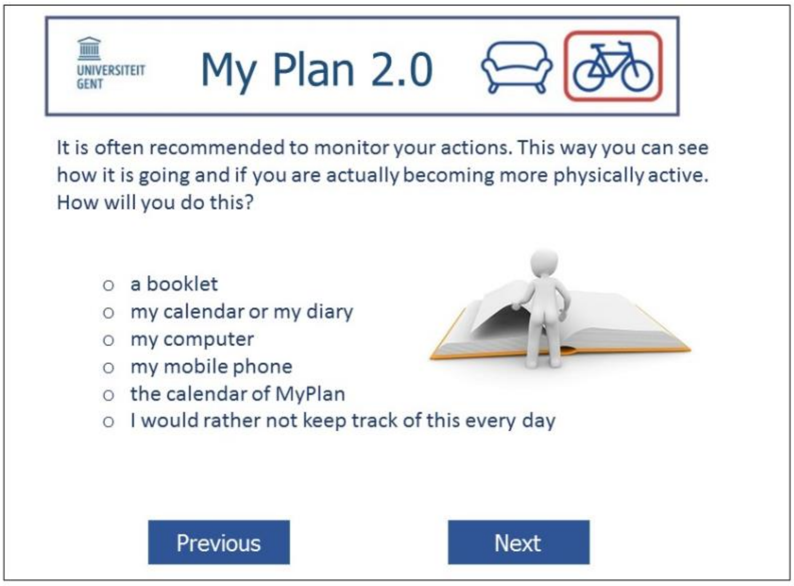

Prompting self-monitoring: Participants can choose how they will self-monitor their behaviour.

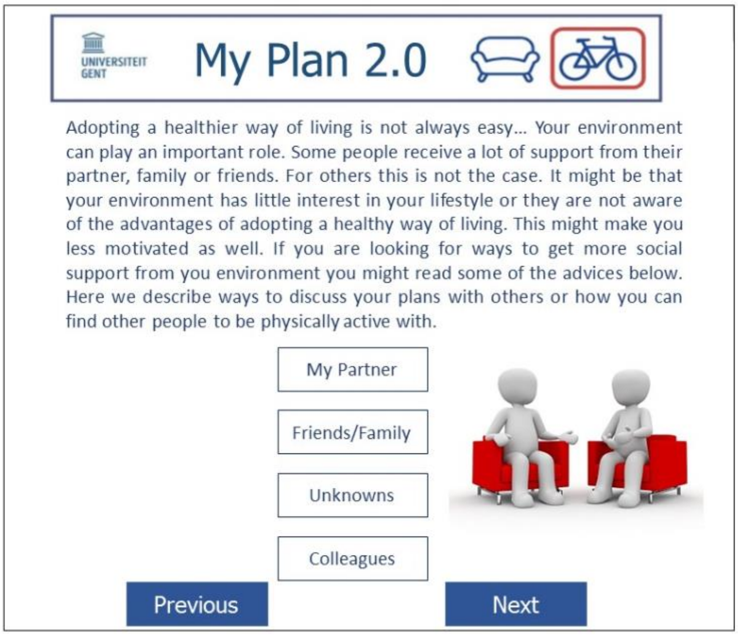

Planning social support: Participants read about how to elicit social support, how to talk about behaviour change to others, and how to engage in behaviour change together with other people.
